# Extral Genital Chancre

**Published:** 1881-12

**Authors:** 


### EXTRA-GENITAL CHANCRE.

A case of syphilis in which the initial lesion occurred on the nose, is reported in the St. Louis Courier of Medicine, (*Am. Specialist*, Nov., 1881). The disease was supposed to have been communicated by a kiss. The husband of the woman was examined and found to have syphilis. The couple had only recently been married and denied any improper relations previous to marriage. Examination of the patient's genital organs showed no sign of any primary lesion ever having existed there. The occurrence of the primary lesion in the locality where it was found was accounted for by the existence of mucous patches in the intended husband's mouth, and the assumption that infected saliva was conveyed to an abraded surface on the nose by kissing. The constitutional manifestations were well developed in the woman, and she aborted in the seventh month of pregnancy.

